# Acetylation: a new key to unlock tau’s role in neurodegeneration

**DOI:** 10.1186/alzrt259

**Published:** 2014-05-29

**Authors:** Casey Cook, Jeannette N Stankowski, Yari Carlomagno, Caroline Stetler, Leonard Petrucelli

**Affiliations:** 1 Mayo Clinic, 4500 San Pablo Road, Jacksonville, FL 32224, USA

## Abstract

The identification of tau protein as a major constituent of neurofibrillary tangles spurred considerable effort devoted to identifying and validating pathways through which therapeutics may alleviate tau burden in Alzheimer’s disease and related tauopathies, including chronic traumatic encephalopathy associated with sport- and military-related injuries. Most tau-based therapeutic strategies have previously focused on modulating tau phosphorylation, given that tau species present within neurofibrillary tangles are hyperphosphorylated on a number of different residues. However, the recent discovery that tau is modified by acetylation necessitates additional research to provide greater mechanistic insight into the spectrum of physiological consequences of tau acetylation, which may hold promise as a novel therapeutic target. In this review, we discuss recent findings evaluating tau acetylation in the context of previously accepted notions regarding tau biology and pathophysiology. We also examine the evidence demonstrating the neuroprotective and beneficial consequences of inhibiting histone deacetylase (HDAC)6, a tau deacetylase, including its effect on microtubule stabilization. We also discuss the rationale for pharmacologically modulating HDAC6 in tau-based pathologies as a novel therapeutic strategy.

## Introduction

The identification of tubulin as the first cytosolic protein to be modified by acetylation [1,2] challenged the traditional notion that acetylation only serves as a mechanism to regulate transcription through modification of histones. Since this discovery in 1985, researchers have sought to identify other proteins that undergo acetylation events and elucidate the effects of this post-translational modification on protein structure and function. Global proteomic studies allowed for the identification of hundreds of proteins that are acetylated on one or multiple lysine residues, as well as a myriad of lysine acetyltransferases and deacetylases, which respectively govern protein acetylation and deacetylation [1,3]. The discovery that the microtubule-associated protein tau is also a target of acetyltransferase and deacetylase enzymes [4,5] added a new layer of complexity, whereby the impact of phosphorylation or ubiquitination on tau function and biology will now need to be re-evaluated to include consideration of tau acetylation. The purpose of the current review is to discuss the recent findings associated with tau acetylation, a novel post-translational modification of tau, how it influences tau aggregation and function, and whether it could be exploited therapeutically as a treatment for tauopathies.

## The impact of tau acetylation on its propensity to aggregate

As lysine residues are unique in their ability to participate in electrostatic and hydrophobic interactions [6,7], and are also known to play a critical role in tau assembly and toxicity [8-10], we and others recently questioned whether tau acetylation of lysine residues would modulate its potential to aggregate [4,11]. Cohen and collagues [4] utilized the acetyltransferase CREB-binding protein (CBP) to acetylate a fragment of tau comprising the microtubule-binding domain (commonly referred to as K18), and observed an increase in aggregation of the K18 fragment. We subsequently performed a similar analysis but using full-length tau and the acetyltransferase p300; we detected a decrease in filament assembly following tau acetylation, the extent of which correlated with the concentration of p300 [11]. We also observed a complete reversal of p300-mediated acetylation and inhibition of tau assembly upon addition of the deacetylase histone deacetylase (HDAC)6 [11]. Furthermore, the modulation of tau assembly by acetylation was dependent on modification of tau’s KXGS motifs in the microtubule-binding domain, as evidenced by the fact pseudoacetylation of the four KXGS motifs generated a tau species that was assembly-incompetent and resistant to modulation by either p300 or HDAC6 [11]. The results from these two studies suggest that CBP and p300 may preferentially acetylate different residues in tau, thereby differentially impacting tau’s intrinsic propensity to aggregate.

Cohen and colleagues [12] later reported that tau can be acetylated in the absence of the enzyme CBP, an effect attributed to a previously unrecognized role of tau as an acetyltransferase enzyme. Cys291 and Cys322 were identified as the catalytic residues responsible for this novel function of tau [12]. We have not observed acetylation of full-length tau in the absence of an exogenous acetyltransferase enzyme [11], indicating that certain experimental conditions, but not all, favor nonenzymatic acetylation [4,12-14]. It is worth noting that nonenzymatic acetylation of cysteine residues has been reported [15], raising the possibility that the increase in tau assembly following acetylation observed by the Cohen group could be due to the modification of amino acid residues other than lysine. Future studies to delineate the physiological consequences of tau acetylation in a site-specific manner and to map the pattern of acetylation produced by different acetyltransferase and deacetylase enzymes are therefore imperative.

## Interplay between post-translational modifications on tau

The multitude of molecular and functional properties of the microtubule-associated protein tau are predominantly due to the protein’s natively unfolded structure, allowing tau to not only interact with a large number of other cellular proteins, but also undergo a variety of post-translational modifications [16]. The occurrence of several post-translational modifications on numerous proteins has been well described, and it has been postulated that the interaction of such modifications governs complex regulatory processes, which are essential for proper protein function and for the regulation of diverse cellular events [3]. While each post-translational modification is distinct and utilizes different chemical groups to modify a given protein on specific residues, a certain degree of overlap exists [3,17]. For instance, lysine residues are targets for acetylation events and other modifications, including ubiquitination, sumoylation and methylation [3]. Thus, a measure of rivalry between different post-translational modifications must exist, where the addition of one chemical group to a given residue precludes further modifications [3].

**Figure 1:**
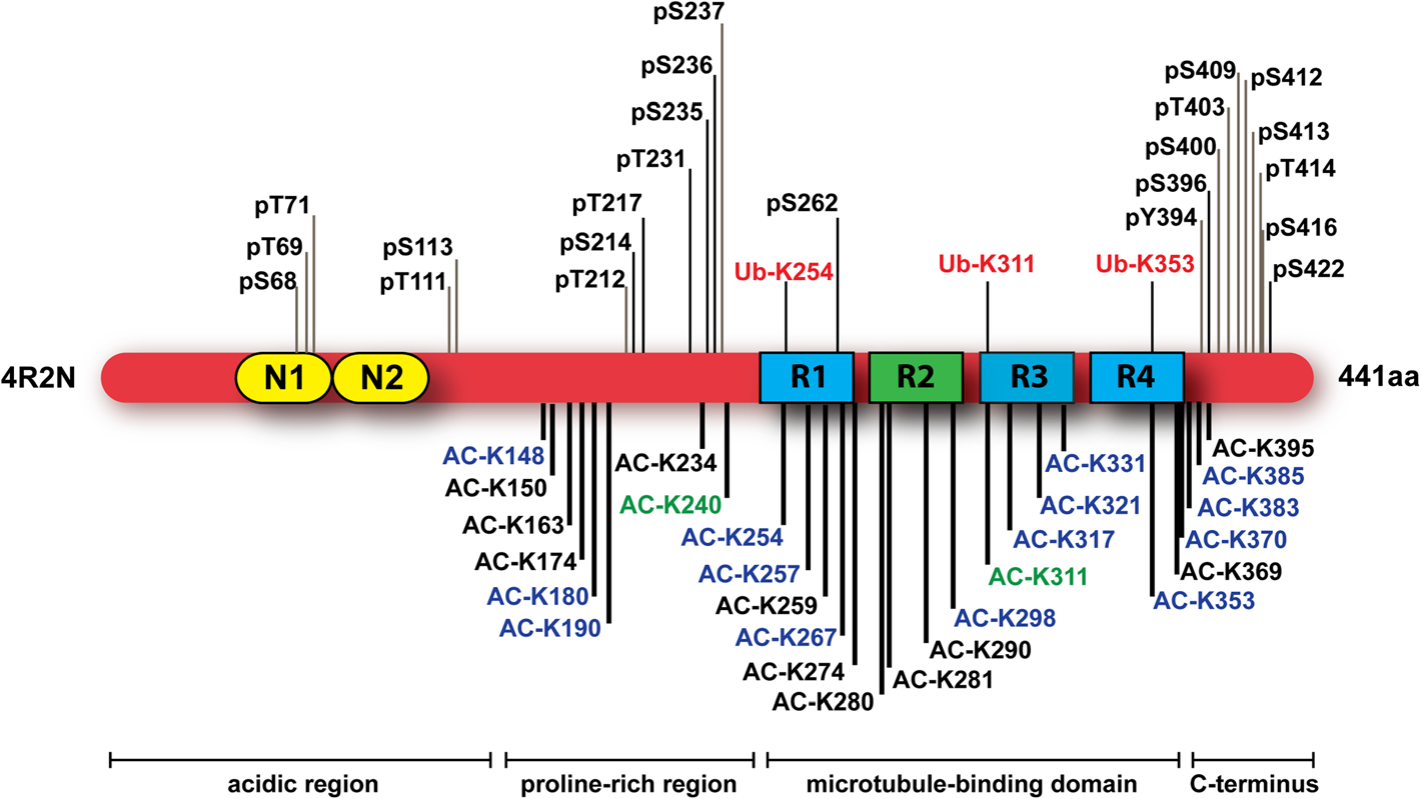
**Schematic diagram of the tau protein with post-translational modification sites.** The molecular domains of the longest isoform of tau (4R2N) are depicted, which includes two amino-terminal inserts (N1 and N2) and four microtubule-binding domain repeats (R1 to R4). The post-translational modifications above the tau molecule depict phosphorylation (black font) and ubiquitination (red font) sites observed in pathological tau species purified from Alzheimer’s disease brain [38]. The post-translational modifications below the tau molecule illustrate the residues that are acetylated by both p300 and CREB-binding protein (black font) [4,5], only p300 (blue font) [5], or only CREB-binding protein (green font) [4].

Intense investigation into the role of post-translational modifications, specifically phosphorylation and acetylation, has now begun, driven by the potential implications of these modifications in Alzheimer’s disease (AD) and other tauopathies [4,11,16,18,19] (Figure 1). Tau’s function as a phosphoprotein is attributed to its 85 potential phosphorylation sites, which are predominantly located in the proline-rich domain and the carboxy-terminal region of the protein flanking tau’s microtubule-binding domains [18,19]. Of these sites, approximately 20 serine and threonine residues have been found to be associated with normal, physiologically important phosphorylation events [20,21]. In addition, it is well-documented that the phosphorylation status of tau is developmentally regulated, with higher phosphorylation levels being present during early developmental stages of the brain compared to the mature, adult brain [16,19,22]. These findings imply that tau phosphorylation is a dynamic, highly regulated process, requiring the precise interplay of a multitude of kinases and phosphatases [18,22]. Because hyperphosphorylation of tau stimulates polymerization and accumulation in the form of insoluble neurofibrillary tangles (NFTs) [18,22,23], research has primarily focused on elucidating the underlying cause of aberrant tau phosphorylation and the effects of this post-translational modification on tau function. While the precise mechanisms underlying the formation of these characteristic neuropathological lesions remain to be fully elucidated, studies have implicated dysregulation of the many kinases and phosphatases that govern tau phosphorylation [16,18,22]. For example, all six isoforms of human tau are constituents of paired helical filaments (PHFs), with all isoforms abnormally hyperphosphorylated [18] in post-mortem brain tissue from AD patients. In addition, tau hyperphosphorylation has been reported to lead to conformational changes that decrease flexibility and affinity for microtubules, thereby promoting accumulation of tau in the cytosol and driving the formation of PHFs and NFTs [18,24]. The polymerization and accumulation of hyperphosphorylated tau (p-tau) has also been linked with impaired axonal transport and synaptic dysfunction, two early events associated with the neuronal degeneration observed in AD and other tauopathies [18,25]. Understanding the precise molecular mechanisms underlying this pathological alteration of tau is critical to identify novel and effective neurotherapeutics for the treatment of AD and other tauopathies.

Acetylation as a novel post-translational modification of tau was first described by Min and colleagues [5], who used synthetic peptides spanning amino acids 160 to 182 and 264 to 287 of the full-length (4R2N isoform) tau sequence to generate acetylated-tau antibodies. With these antibodies (referred to as Ab708 and 9AB), sirtuin 1 (SIRT1) was identified as the deacetylase for the acetylation sites contained within amino acids 160 to 182 (Ab708) and 264 to 287 (9AB) [5]. Based on these results, the authors proposed a model whereby the deacetylase activity of SIRT1 promotes the removal of acetyl groups from tau, thus allowing for the addition of ubiquitin to these freed lysine residues and subsequently enhancing tau’s proteasomal turnover [5]. This model speaks to the previously addressed complexity associated with post-translational modifications of tau, and underscores that we are still at the beginning of understanding which enzymes regulate tau acetylation and deacetylation events, and more specifically how tau modifications associated with acetylation govern tau biology.

The discovery that tau is modified by acetylation [5] has since prompted new research efforts primarily focused on assessing the pathological significance of this novel aspect of tau biology [4,5,26,27]. Specifically, Irwin and associates [27] generated a novel antibody detecting acetylation of tau at K280, and concluded that tau acetylated at this epitope colocalized with other classical markers of tau pathology, with colocalization being most prominent in moderate to severe disease stages. Given that acetylation of tau at K280 is highest at late stages of disease, it is possible increased acetylation at this epitope is a response to the disease process, rather than a pathogenic mechanism responsible for tangle formation. In a subsequent report, Grinberg and colleagues [26] focused on a different tau acetylation site, and developed an antibody to detect acetylation on K274. Although enhanced acetylation of tau on this epitope was observed in most tauopathies, the authors detected a lack of tau acetylation on K274 in the tauopathy agyrophilic grain disease [26]. As such, it is evident that additional research evaluating tau acetylation is needed to elucidate differences that may be attributed to site and disease.

The discovery of tau acetylation also prompted efforts in our own lab to elucidate whether HDAC6 activity modulates tau’s pathogenicity directly through tau deacetylation [11,28]. We demonstrate that elevated HDAC6 activity increases phosphorylation of tau at the 12E8 epitope (pS262/356), a phospho-epitope present within the KXGS motifs of tau’s microtubule-binding domain. The phosphorylation of KXGS motifs within tau by the kinase Par-1/MARK2 is required for tau proteotoxicity in *Drosophila *[29], observed at very early stages of NFT formation in AD brain [30], and appears to prime tau for subsequent phosphorylation events [29,31]. Tau species phosphorylated on KXGS motifs are not recognized by cellular degradation machinery, including the tau ubiquitin ligase C-terminus of Hsc70 interacting protein (CHIP) and the heat shock protein 70/90 (Hsp70/90) chaperone complex [32,33], and are therefore particularly prone to accumulate. Of note, the synaptic toxicity of oligomeric amyloid beta is dependent upon the phosphorylation of tau’s KXGS motifs [34,35], providing additional support for a pathogenic role of this phospho-tau species. Given that tau species phosphorylated on KXGS motifs are resistant to degradation and accumulate in NFTs [30], fail to bind and stabilize microtubules [36], and are also primed for phosphorylation by other kinases [29,31], hyperactivation of HDAC6 would be expected to considerably enhance tau pathology. We recently demonstrated that, in addition to modulating phosphorylation at these critical KXGS motif regions, HDAC6 also regulates their acetylation [11]. As mentioned above, this acetylation decreases the ability of tau to aggregate in *in vitro* assays [11]. We also detect a competitive relationship between acetylation and phosphorylation on KXGS motifs, such that treatment with HDAC6 inhibitors simultaneously increases acetylation of tau, while blocking phosphorylation at these crucial motifs in mice [11]. Furthermore, KXGS motifs are hypoacetylated and hyperphosphorylated in patients with AD and in a progressive and well-characterized mouse model of tauopathy (rTg4510) [11,37]. The link between a loss of acetylation on KXGS motifs and disease pathogenesis is further strengthened by the observation that KXGS motifs are ubiquitinated in pathological tau purified from post-mortem human brain tissue in AD [38] (Figure 1), thereby indicating such ubiquitination would preclude another post-translational modification, acetylation, from occurring.

Overall, these results highlight the extreme complexity of post-translational modifications governing tau function, and illustrate the need for improved understanding of how modified tau species impact neuronal health. Given that HDAC6 had no effect on the acetylation of residues recognized by the Ab708 antibody [5], and that we recently demonstrated that deacetylation of KXGS motifs is mediated by HDAC6 and not SIRT1 [11], the pattern of tau acetylation is determined by more than one deacetylase. Future studies will be required to map the specific acetylation sites regulated by different deacetylases in order to determine the ultimate consequence(s) of modulating deacetylase (HDAC6 versus SIRT1) activity on tau function and biology. Moreover, pathological alterations of tau are most likely not the result of a single post-translational modification, but instead a combination of structural and functional alterations that may ultimately contribute to determine tau pathogenicity.

## Effect of acetylation on tau turnover

Following identification of the tau protein as a major constituent of NFTs in AD and other tauopathies, several lines of research focused on identifying the mechanism(s) responsible for tau accumulation in disease. Most conducted studies have focused on the effect of hyperphosphorylation on tau turnover, due to the fact that hyperphosphorylation has been the first and one of the most prominent post-translational modifications associated with tau pathology [33,39-42]. In particular, previous studies have demonstrated that the tau ubiquitin ligase, CHIP, is unable to bind and ubiquitinate tau species phosphorylated by Par-1/MARK2 on the 12E8 epitope (S262/356) [33], a p-tau species that is also resistant to degradation upon treatment with Hsp90 inhibitors [32,33]. Tau phosphorylated at the PHF1 epitope (S396/404) is still susceptible to degradation following Hsp90 inhibition and actually exhibits an enhanced interaction with Hsp90 [33]. These findings indicate that certain p-tau species, rather than normal tau, are a preferred client protein of Hsp90, while some phosphorylation events, in particular those mediated by Par-1/MARK2 on tau’s KXGS motifs, generate a p-tau species not recognized by the chaperone network. Phosphorylation by Par-1/MARK2 on KXGS motifs in the microtubule-binding domain of tau has been shown to be required for initiation of the pathogenic cascade of hyperphosphorylation, which is ultimately associated with NFT formation in tauopathies [29]. HDAC6 disrupts this cascade by potentiating Par-1/MARK2-mediated phosphorylation of KXGS motifs (detected by the 12E8 antibody), an effect that is eliminated by pseudoacetylation of KXGS motifs [11]. In addition, our recent findings indicate that HDAC6 directly modulates tau polymerization and acetylation, and this relationship is dependent on the ability of HDAC6 to deacetylate tau specifically on KXGS motifs [11]. These results support the hypothesis that decreased HDAC6 activity increases acetylation of KXGS motifs and, in so doing, prevents phosphorylation of serine residues within the same motif. As acetylation and phosphorylation of KXGS motifs act in a competitive manner, and phosphorylation of KXGS motifs generates a p-tau species that is resistant to degradation, future studies will be required to determine whether acetylation of tau on KXGS motifs impacts the ability of the chaperone network to recognize tau in a similar manner to phosphorylation on these sites. Given that progressive hypoacetylation and hyperphosphorylation of KXGS motifs is observed in rTg4510 mice with aging [11], the fact that tau turnover also decreases with aging in rTg4510 mice [43] may indicate that the relationship between acetylation and phosphorylation on KXGS motifs regulates tau turnover. The effects of other post-translational modifications on tau turnover are unknown; thus, it remains to be determined whether differentially modified tau species are degraded by the same mechanisms as hyperphosphorylated tau, or if they are preferentially targeted to alternative degradation pathways.

While hyperphosphorylated tau is ubiquitinated in patients with AD [38,44] (Figure 1), indicating that pathological tau species may be successfully targeted for degradation, the accumulation of ubiquitinated tau species in those patients suggests dysfunction of either proteasomal or lysosomal degradation pathways contributes to NFT formation in disease. The fact that ubiquitination and acetylation both modify lysine residues indicates that these post-translational modifications most likely compete to modify specific residues. Given that aggregated tau within NFTs is ubiquitinated [38,44], it is possible that excessive ubiquitination of tau actually prevents acetylation, exacerbating tau aggregation. The notion that excessive ubiquitination of tau may be detrimental in tauopathies is somewhat counterintuitive. However, as NFTs are composed of ubiquitinated tau, it is clear that a failure of the cell to ubiquitinate tau is not the root cause of tau accumulation. Thus, strategies to further enhance ubiquitination of tau are not likely to promote tau clearance in disease, indicating that different and potentially unconventional approaches will need to be considered in designing therapeutic strategies of the future. Specifically, recent evidence indicates that acetylation of tau on KXGS motifs under conditions of HDAC6 inhibition not only prevents aggregation, but also blocks phosphorylation on this same motif, thereby favoring tau clearance [11]. In addition, reports that KXGS motifs in tau species purified from NFTs are ubiquitinated [38,42] indicate that these KXGS motifs are not acetylated, consistent with the notion that tau species modified by acetylation on KXGS motifs do not aggregate into NFTs. As such, rather than developing strategies to enhance tau ubiquitination in an effort to facilitate clearance, strategies to promote tau acetylation specifically on KXGS motifs could be evaluated for therapeutic efficacy.

## Loss of HDAC6 alleviates defects in tau and amyloid precursor protein models

Based on recent evidence that HDAC6 regulates tau acetylation on KXGS motifs, it is of particular interest that, in a *Drosophila* model of tauopathy, loss of HDAC6 activity rescued tau-induced microtubule defects in both neuronal and muscle cells [45]. This finding provides the first *in vivo* evidence that reducing HDAC6 activity in a model of tauopathy is protective. Further emphasizing the therapeutic potential of HDAC6 inhibitors are results demonstrating that loss of HDAC6 expression/activity is also neuroprotective in other neurodegenerative diseases, including AD, Huntington’s disease and amyotrophic lateral sclerosis [46-48]. For instance, in a mouse model of AD, genetic ablation of HDAC6 alleviated cognitive impairment without impacting plaque burden, which may suggest that beneficial consequences of loss of HDAC6 expression are due to effects on endogenous tau, though this has not yet been assessed in this model [47]. Deletion of HDAC6 in a mouse model of mutant superoxide dismutase 1-linked amyotrophic lateral sclerosis is also neuroprotective, as reflected by the extended life span of mice and increased motor axon integrity [48].

Several groups have demonstrated that loss of HDAC6 activity rescues impaired mitochondrial trafficking along microtubules [47,49,50], most likely through enhanced tubulin acetylation, providing additional insight into the mechanisms by which HDAC6 inhibition enhances neuronal survival. In particular, tubulin acetylation has been shown to enhance the recruitment of molecular motors kinesin-1 and dynein [46], thus facilitating anterograde and retrograde transport along the microtubular network [50,51]. The decrease in tubulin acetylation and increased HDAC6 observed in patients with AD and other tauopathies is indicative of a disrupted microtubular network, which would be expected to contribute to the pathophysiological changes associated with disease progression [50,52,53]. Another recent report identified a decrease in microtubule stability in rTg4510 mice [54], and also verified that treatment with the microtubule-stabilizing compound epothilone D (EpoD) decreased tau burden and cognitive deficits [54]. Zhang and colleagues [55] also reported that treatment with EpoD not only effectively decreased tau pathology in another tau transgenic mouse model (PS19 mice), but also increased axonal microtubular density. The improvement of microtubular stability by EpoD subsequently resulted in improved axonal transport and cognitive performance as assessed by a battery of behavioral tests [55]. Given that HDAC6 inhibition similarly augments axonal transport through enhanced tubulin acetylation, these findings suggest that reduced HDAC6 activity would also decrease tau burden and cognitive deficits in tauopathy.

The coordinated regulation of HDAC6-mediated tubulin acetylation and tau acetylation on KXGS motifs may allow for tight regulation of microtubule dynamics and axonal transport. While tubulin acetylation is a marker of microtubule stability [56], increased tau acetylation may allow tau to dissociate from stabilized microtubules, providing molecular motors greater access to microtubules and facilitating axonal transport. The dissociation of tau and tubulin under conditions of enhanced microtubule stability is supported by a recent study employing FRET technology and live cell imaging to monitor the tau/tubulin interaction [57]. Conversely, conditions of heightened HDAC6 activity presumably lead to enhanced deacetylation of both tubulin and tau, which may promote tau-microtubule interactions, leading to increased microtubule stability. Given that phosphorylation, which prevents acetylation, within KXGS motifs has also been reported to release tau from microtubules [36], this event would be expected to uncouple the coordinated regulation of tubulin and tau acetylation, further contributing to the pathogenicity of this particular p-tau species. Overall, these results further speak to the complexity associated with the biology of the tau protein and underline how minute, molecular dysfunctions can contribute to the tau pathology observed in patients with AD and other tauopathies. Moreover, these studies also highlight the pressing need to better understand tau biology under physiological as well as pathological conditions.

## Tau acetylation: implications on propagation

There is now considerable evidence supporting the trans-cellular propagation and seeding of tau pathology in a variety of *in vitro* and *in vivo* models, ultimately demonstrating that extracellular tau filaments can be internalized by cells and function as seeds for the assembly of intracellular filaments [58-63]. While the precise mechanism(s) underlying trans-neuronal tau propagation has yet to be elucidated, recent work is beginning to provide insight into this pathway. Wu and colleagues [64] observe internalization of misfolded tau at the level of both dendritic and axonal terminals in neurons, after which pathologic tau species can be transported in either the antero- or retrograde direction, thereby leading to the spreading of pathology. In addition, injection of brain material from mice that express human mutant P301S tau into transgenic mice expressing human wild-type tau (ALZ17 model) was sufficient to induce tau pathology not only within, but also adjacent to, the injection site along anatomically connected pathways [58]. Furthermore, injection of brain extracts from patients with different tauopathies into either ALZ17 or non-transgenic mice was not only sufficient to drive inclusion formation, but actually effectively reproduced the classic hallmark lesions of the specific tauopathy characteristic of the inoculating brain extract [65]. These studies provide additional support for the concept that pathologically altered tau species possess a remarkable self-propagating and seeding capacity, and also indicate that seeding-competent tau species are somehow different and distinct across the class of tauopathies, such that the inoculating material acts as an exact template in the new host. The specific characteristics of pathological tau species that define and determine seeding capacity remain to be identified, and could be the result of a precise pattern of post-translational modifications that differentially impact conformation of the tau molecule and ultimately determine aggregate structure. Our recent findings, which demonstrate that acetylation within tau’s KXGS motifs generates a tau species that fails to polymerize [11], suggests that augmenting acetylation of the KXGS motifs would also decrease tau seeding capacity.

## Conclusion

We review here the rationale supporting the utilization of HDAC6 inhibition to enhance tau acetylation as a novel therapeutic strategy for tauopathies. HDAC6 inhibitors simultaneously promote acetylation and prevent phosphorylation of tau on KXGS motifs, thereby interfering with tau’s propensity to aggregate. Decreasing HDAC6 activity also enhances microtubule stability and transport, which is expected to further stimulate neuronal function. As HDAC6 inhibitors are currently being evaluated in clinical trials for oncology indications, data will soon be available to assess the safety of pharmacologic modulation of HDAC6 in humans, which could expedite their repurposing for other diseases. Although additional research is needed to fully elucidate the cellular and molecular pathways associated with the neuroprotective consequences of HDAC6 inhibition, it is becoming increasingly apparent that modulating HDAC6 activity may offer a very promising avenue for the treatment of AD and associated tauopathies.

## Note

This article is part of a series on *Tau-based therapeutic strategies*, edited by Leonard Petrucelli. Other articles in this series can be found at http://alzres.com/series/tau_therapeutics.

## Abbreviations

AD: Alzheimer’s disease; CBP: CREB-binding protein; CHIP: C-terminus of Hsc70 interacting protein; EpoD: Epothilone D; HDAC: Histone deacetylase; Hsp: Heat shock protein; NFT: Neurofibrillary tangle; PHF: Paired helical filament; p-tau: Hyperphoshorylated tau; SIRT1: Sirtuin 1.

## Competing interests

The authors declare that they have no competing interests.
